# Multiplicity and diversity of *Plasmodium falciparum* gametocytes

**DOI:** 10.1186/1475-2875-11-S1-P100

**Published:** 2012-10-15

**Authors:** Rahel Wampfler, Lincoln Timinao, Ivo Mueller, Ingrid Felger

**Affiliations:** 1 Swiss Tropical and Public Health Institute, Basel, Switzerland; 2 Papua New Guinea Institute of Medical Research, Goroka, Papua New Guinea; 3 Walter and Eliza Hall Institute, Melbourne, Australia

## Background

Discrimination of gametocyte-producing *P. falciparum* clones depends on high expression of one or more polymorphic stage-specific markers and on the genetic diversity of these markers in the study area. *Pfs230* and *pfg377* are classical length-polymorphic markers for differentiation of gametocytes. Because of variable PCR fragment sizes, these markers are particularly well suited to distinguish gametocytes of multiple *P. falciparum* clones within a patient. We aimed at improving the resolution of both markers by creating amplicons spanning several polymorphic domains of these genes and by increasing the sizing accuracy by capillary electrophoresis using an automated sequencer.

## Material and methods

We assessed the genetic diversity and the multiplicity of *pfs230* and *pfg377* in 80 DNA samples from Papua New Guinea by nested-PCR and following sizing by capillary electrophoresis. We also investigated novel size-polymorphic gametocyte markers, such as *PF11.1* (*PF10_0374*), *PF11_0214*, *PFI0205w* and *PFL0105w*, as well as SNP-based genotyping approaches.

## Results

We observed high diversity with *pfs230* (He=96.3) and *pfg377* (He=89.4). 17 and 13 different alleles were found for *pfs230* and *pfg377*, respectively. The multiplicity of infection (MOI) of *pfs230* and *pfg377* was compared with the asexual MOI by marker *msp2* for each sample.

## Discussion

Gametocyte typing of field samples requires RNA sampling and high gametocyte-specific expression of the genotyping marker. A gametocyte trendline was used to evaluate the detection limit of the nested-RT-PCR of *pfs230* and *pfg377* in comparison to the standard marker for gametocyte detection, *pfs25.* We discuss the application of high-resolution gametocyte genotyping for studies on malaria transmission.

